# Spatial transcriptomics and neurofilament light chain reveal changes in lesion patterns in murine autoimmune neuroinflammation

**DOI:** 10.1186/s12974-023-02947-y

**Published:** 2023-11-13

**Authors:** Tobias Brummer, Miriam Schillner, Falk Steffen, Flores Kneilmann, Beatrice Wasser, Timo Uphaus, Frauke Zipp, Stefan Bittner

**Affiliations:** Department of Neurology, Focus Program Translational Neuroscience (FTN) and Immunotherapy (FZI), Rhine Main Neuroscience Network (Rmn2), University Medical Center of the Johannes Gutenberg University Mainz, Langenbeckstr. 1, 55131 Mainz, Germany

**Keywords:** Multiple sclerosis, Experimental autoimmune encephalomyelitis, Serum neurofilament, Microglia, Spatial transcriptomics

## Abstract

**Objective:**

Ongoing neuroaxonal damage is a major contributor to disease progression and long-term disability in multiple sclerosis. However, spatio-temporal distribution and pathophysiological mechanisms of neuroaxonal damage during acute relapses and later chronic disease stages remain poorly understood.

**Methods:**

Here, we applied immunohistochemistry, single-molecule array, spatial transcriptomics, and microglia/axon co-cultures to gain insight into spatio-temporal neuroaxonal damage in experimental autoimmune encephalomyelitis (EAE).

**Results:**

Association of spinal cord white matter lesions and blood-based neurofilament light (sNfL) levels revealed a distinct, stage-dependent anatomical pattern of neuroaxonal damage: in chronic EAE, sNfL levels were predominately associated with anterolateral lumbar lesions, whereas in early EAE sNfL showed no correlation with lesions in any anatomical location. Furthermore, neuroaxonal damage in late EAE was largely confined to white matter lesions but showed a widespread distribution in early EAE. Following this pattern of neuroaxonal damage, spatial transcriptomics revealed a widespread cyto- and chemokine response at early disease stages, whereas late EAE was characterized by a prominent glial cell accumulation in white matter lesions. These findings were corroborated by immunohistochemistry and microglia/axon co-cultures, which further revealed a strong association between CNS myeloid cell activation and neuroaxonal damage both in vivo and in vitro.

**Interpretation:**

Our findings indicate that CNS myeloid cells may play a crucial role in driving neuroaxonal damage in EAE. Moreover, neuroaxonal damage can progress in a stage-dependent centripetal manner, transitioning from normal-appearing white matter to focal white matter lesions. These insights may contribute to a better understanding of neurodegeneration and elevated sNfL levels observed in multiple sclerosis patients at different disease stages.

**Graphical Abstract:**

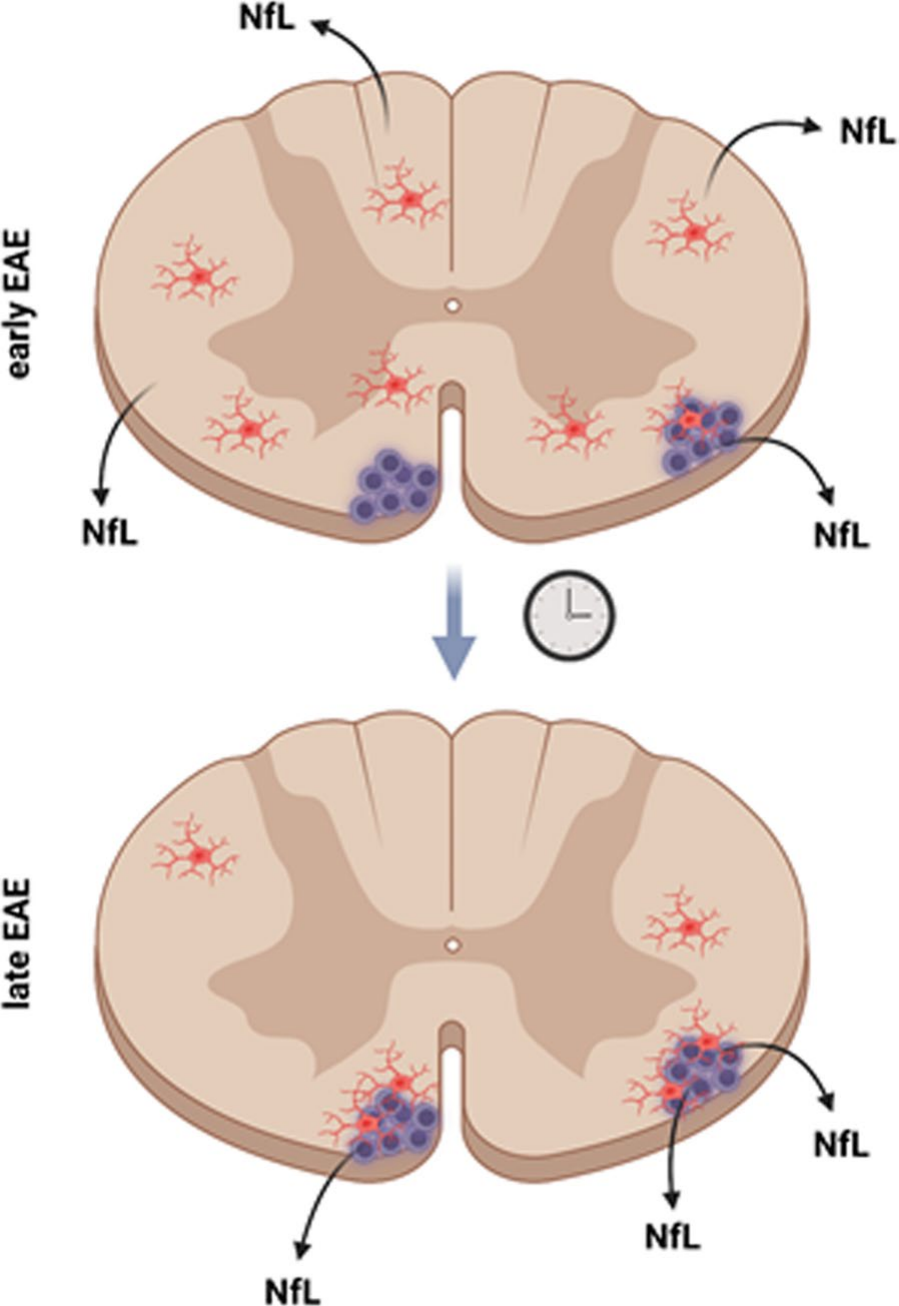

**Supplementary Information:**

The online version contains supplementary material available at 10.1186/s12974-023-02947-y.

## Introduction

Multiple sclerosis (MS) is a chronic inflammatory CNS disorder in which disability progression is closely related to ongoing neuroaxonal degeneration. Thus, detection of axonal damage as well as its quantification is a crucial step towards improved clinical decision-making and therapy stratification. Recently, technical advances such as single-molecule array (SiMoA) have enabled the detection of low abundant proteins in blood samples, abolishing the need for more invasive sampling methods, such as lumbar puncture [1]. Therefore, serum-based neurofilament light chain (sNfL) has gained vast diagnostic interest and is on the cusp of being implemented in clinical routine diagnostics [1, 2]. NfL is part of the neuronal intermediate filaments, which constitute a major portion of the axonal cytoskeleton [3, 4]. Upon axonal injury, neurofilaments are released into the extracellular space and ultimately into the blood, where they can serve as surrogate markers of neurodegeneration (independent from the underlying etiology) [1, 4].

Little is known about the exact pathophysiological aspects of NfL release from damaged axons. Furthermore, it has never been systematically addressed whether the magnitude of NfL release is dependent on the respective anatomical location of inflammatory CNS lesions at different disease stages. Specifically, it is unclear to which extent axonal damage in the normal-appearing white matter (NAWM) contributes to the total NfL release.

People with MS (pwMS) generally display a high inter-individual variance of sNfL levels [1], which may be explained by differences in the anatomical location of white matter lesions (WML). In line with this, clinical severity scores—like the expanded disability status scale (EDSS)—and sNfL levels only correlate moderately with the total lesion burden [1], suggesting that certain strategic lesions may result in low NfL levels, but severe (usually motor) disability and vice versa. The abundance of NfL is greater in longer, thicker axonal tracts than in thinner, shorter axons [3, 4], thus damage to these structures could result in higher sNfL levels. This may be particularly relevant for infratentorial lesions, which predominately affect long, tightly packed axonal tracts [5]. Myelin oligodendrocyte glycoprotein (MOG)-induced experimental autoimmune encephalomyelitis (EAE) is a rodent model of MS, which predominately manifests in the spinal cord (SC) [6]. Hence, MOG-EAE may represent an appropriate model to study damage to long, ascending and descending infratentorial axonal tracts. Many recent studies have highlighted the involvement of CNS-myeloid cells (CMC)—such as microglia and invading macrophages—in disease progression and axonal damage in pwMS [7, 8]. Promising therapeutic strategies include hydroxychloroquine [9] and Bruton's tyrosine kinase (BTK) inhibitors, for which there are several ongoing phase III trials in MS. Despite these promising developments, the association of CMC and spatio-temporal neuroaxonal degeneration/NfL release remains incompletely understood.

Here, we show that spatio-temporal sNfL release in EAE can be driven by CMC and may progress in a disease stage-dependent centripetal manner from NAWM to WML. Furthermore, we demonstrate that sNfL levels are associated with the respective anatomical lesion location and therefore offer a pathophysiological explanation for the high inter-individual variance of sNfL levels in pwMS.

## Methods

### Animals

C57BL/6J (B6) mice were purchased from Janvier Labs (Saint-Berthevin Cedex, France). Mice were housed in the Translational Animal Research Center of the University Medicine Mainz under a 12 h/12 h light/dark cycle and had access to water and food ad libitum. Animal procedures were performed under the supervision of authorized investigators in accordance with the European Union normative for care and use of experimental animals, conducted according to the German Animal Protection Law, and approved by the appropriate state committees for animal welfare (Landesuntersuchungsamt Rheinland-Pfalz, TVA# 23 177-07/G20-1-074).

### Induction of EAE

Induction procedures were performed as previously described [10]. Mice were randomly assigned to either an early (stable score 3 days after peak) or late (stable score 20 days after peak) EAE cohort.

### Collection of blood and CSF

Collection of blood and CSF was performed according to established procedures [11].

### Histology and immunohistochemistry

All procedures were performed as previously described [12]. Cryosections (10 µm) of EAE mouse brains were stained with hematoxylin and eosin (H&E) and luxol fast blue. Pictures were obtained using a Keyence BZ-X710 fluorescence microscope (Keyence) and analyzed with ImageJ software. The following primary antibodies were used: SMI32 (BioLegend; 801701; 1:1000), GFAP (Sigma; G3893; 1:1000), Iba1 (Sigma Aldrich; ab153696; 1:500), CD4 (LS BioScience; LS-C413024; 1:100). SMI32-positive axons as well as Iba1 and glial fibrillary acidic protein (GFAP) stainings were quantified in 200** × **200 µm regions of interest (ROI).

### Anatomical classification of lesions

Lesions were identified by the respective mononuclear infiltrate and classified according to their anatomical location (cervical, thoracic, lumbar, anterolateral and dorsal) and contact to the CSF. Sections above and below each section were checked for CSF contact. If no CSF contact was detected in five consecutive sections, lesions were classified as lesions without CSF contact. Relative lesion sizes per white matter area per section were quantified in a blinded fashion with ImageJ software: *Relative lesion size* = *(Lesion area/(Total area of the section − grey matter area))* × *100%.*

### Western blotting

Western blots were performed and recorded on an Odyssey^®^ Fc Imaging System (LI-COR Biosciences, USA) as previously described [12]. The following primary and secondary antibodies were used: chicken polyclonal to 68 kDa Neurofilament (abcam, UK) with IRDye^®^ 680RD Donkey anti-Chicken (LI-COR Biosciences, USA) and rabbit anti-α/β-Tubulin Antibody (Cell Signaling Technology, USA) with IRDye^®^ 800CW Goat anti-Rabbit IgG.

### NfL measurements with SiMoA and ELISA

SiMoA measurements were performed as described in detail previously [13]. Samples were measured in a blinded fashion in duplicates; the intra-assay coefficient of variation (CV) of all samples was 6.01%. ELISA measurements of cell culture supernatants were performed in accordance with the manufacturer’s instructions (NF-light™ Serum ELISA, Uman Diagnostics, Sweden).

### Microglia–axon co-cultures

Primary cortical neurons were prepared and plated into XONA microfluidic chips (XC450, Xona Microfluidics, USA) as previously described and in accordance with the manufacturer’s instructions [14]. Microglia–axon co-cultures were performed following the protocol of Fujita and Yamashita [15]. Microglia were prepared from adult mice, using the adult brain dissociation kit (Miltenyi Biotec) according to the manufacturer’s instructions. Half of the medium was changed every 2 days. After 7 days in vitro (DIV), microglia were activated by lipopolysaccharide (LPS) (10 ng/ml) or vehicle for 24 h. At 8 DIV, microglia and /or microglia media were harvested, and added to the axonal side of the XONA microfluidic chambers. Co-cultures were incubated for 6 h, media was then collected, cells fixed with 4% PFA and stained with the following primary antibodies: Tuj1 (BioLegend; 801201; 1:500), Iba1 (Sigma Aldrich; ab153696; 1:500).

### Spatial transcriptomics

Mice were killed, SCs were extracted without perfusion and embedded separately in OCT Tissue Tek^®^ (Sakura Finetek), snap frozen in isopentane on dry ice and stored at − 80 °C. The embedded SCs were cut coronally into 10-μm sections using a CryoStar NX70 cryostat (Thermo Fisher Scientific). Sections were checked for the presence of lesions by a short H&E staining. The next section was transferred onto the capture area of a Visium slide (10 × Genomics, Pleasanton CA, USA). Each 6.5** × **6.5 mm capture area contains roughly 5000 spots with primers and spatial barcodes. Visium Spatial Gene Expression was performed following the User Guide (Document CG000239 Rev A). Sectioned tissue was fixed in chilled methanol at − 20 °C for 30 min and H&E stained and imaged as described above. After imaging, slides were placed in a slide cassette and processed using the Visium Gene Expression Reagent Kit (10 × Genomics). Sections were permeabilized for 12 min, followed by reverse transcription, cDNA synthesis and amplification, as well as purification with SPRIselect magnetic beads (Beckman Coulter). Cycle number for amplification was set by determining the Cq value using the KAPA SYBR FAST qPCR Master Mix (KAPA Biosystems) and was between 12 and 16 cycles. Purified cDNA was stored at − 20 °C for up to 4 weeks before cDNA quality control (QC) and library preparation. cDNA QC was performed on an Agilent 2100 Bioanalyzer with a high sensitivity DNA assay (Agilent Technologies) and cDNA yield was determined. Libraries were constructed using the Library Construction Kit (10 × Genomics). After fragmentation, end repair, A-tailing and adaptor ligation, samples were indexed using the Dual Index Plate TT Set A (10 × Genomics) with amplification cycles between 12 and 16 based on the cDNA yield. Double-sided size selection using SPRIselect magnetic beads (Beckman Coulter) was performed and finished libraries were stored at − 20 °C before sequencing. Paired-end sequencing was performed using NovaSeq 6000 (Illumina).

### Analysis of spatial transcriptomics

After manual alignment of the slides in Loupe Browser 6 (10x Genomics), we generated a filtered feature-barcode matrix of all samples (healthy, early and late EAE) with spaceranger (version 1.3.1) count and aggr and performed data analysis in R 4.1.3 with the Seurat 4.1.1 toolkit [16]. Upon discovering a dominant batch effect in the Uniform Manifold Approximation and Projection (UMAP) visualization, we ran Harmony version 0.1.0 [17] to obtain batch-corrected principal component analysis (PCA) embeddings (10 principal components of the scaled-and-centered variable features in log-normalized data). Harmony settings were taken from benchmarking demonstration [18]; additionally, we set cluster width to 0.2, block.size to 0.005, epsilon.cluster to 1e-8 and epsilon.harmony to –Inf. We used the corrected PCA embeddings for graph-based clustering [19] in Seurat with k set to ln(number of spots) like in spaceranger and resolution to 0.5. We added a cluster-merging step based on hierarchical clustering of cluster-centroids in PCA space: a node of clusters was merged if no principal component was able to classify its branches with an absolute (AUC-0.5)*2 over 0.8 (AUC, area under the curve). We annotated the clusters with anatomical areas by cross-referencing to H&E-stained images in Loupe Browser. We performed a subsetting of spots in Seurat, labeling spots as “lesions” if they showed any expression of at least two of CD3, CD4 or CD8. Afterwards, we performed differential gene expression (DGE) testing between EAE early and late conditions in two subsets of the white matter spots: those labeled “lesions” and those not (thus termed “NAWM”). We used MAST [20] in Seurat for DGE testing and incorporated the cellular detection rate as covariate, which is considered the best practice for handling batch effects [21]. Genes were considered significant if their Bonferroni-adjusted *p*-value was below 0.05. We analyzed Gene Ontology (GO) term enrichment against the background of all genes expressed in the feature-barcode matrix with ClusterProfiler [22] and the org.Mm.eg.db mouse annotation. We computed the Jaccard similarities between ‘biological processes’ terms and visualized the resulting network with enrichplot. To get functional annotation modules, we used GO enrichments for the genes significantly regulated in each direction in either of the spot subsets. Then, by manually summarizing clusters in the resulting network plots and redundant or related GO terms, we made 15 modules of immune-, glia- and neuron-related processes, assigning each GO term uniquely to one module. To assign genes to the modules, we used a GO enrichment for the overall list of all significantly regulated genes. Genes were then assigned to the module(s) that contained any enriched terms they contributed to.

### Statistics

All tests were performed on an exploratory two-sided 5% significance level. Appropriate statistical tests were chosen based on the experimental condition. Normal distribution of data was assessed using a corresponding normality test. P-values for pairwise comparisons were calculated with relative values using two-sided Student's t-test or Mann–Whitney U. P-values for comparisons of multiple groups were calculated with one-way analysis of variance (ANOVA) with Dunnett's multiple comparison test for multiple hypothesis testing. Correlations were performed using the Spearman or Pearson rank correlation. Statistical analyses were performed with GraphPad Prism9 software.

## Results

### Anatomical NfL expression and release in murine neuroinflammation

**Fig. 1:**
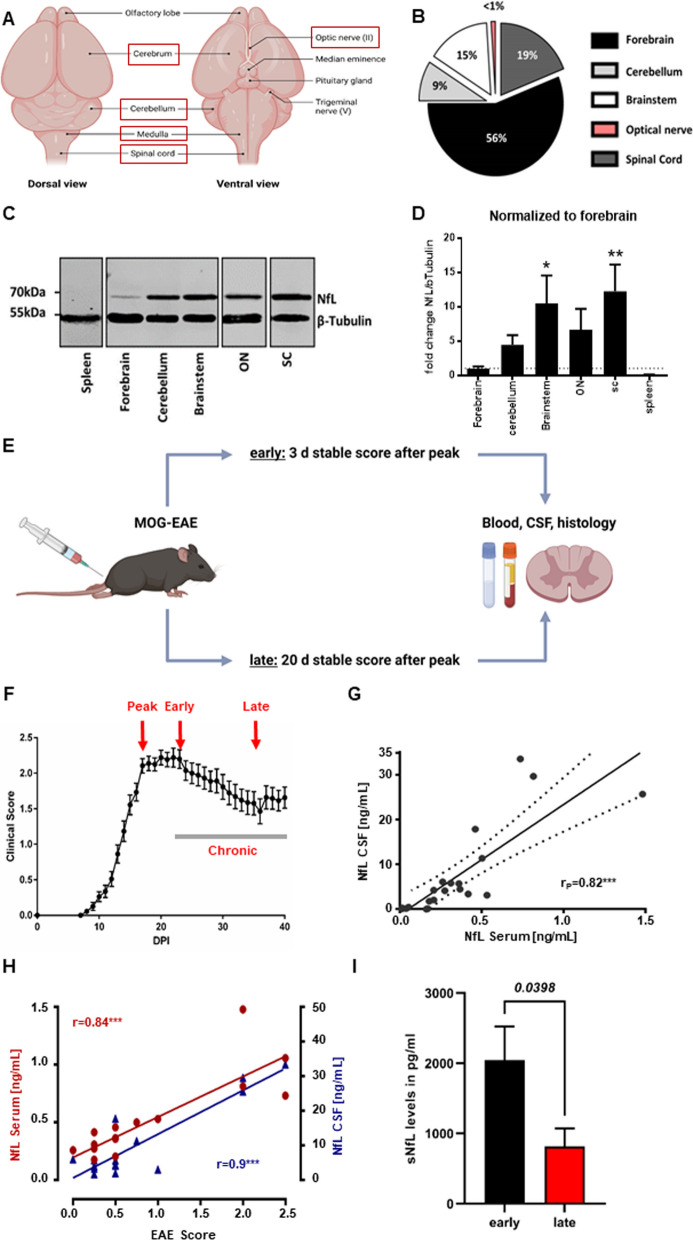
NfL expression in various anatomical locations and NfL release in MOG-EAE.** A** Cartoon depicting the investigated murine brain regions in adult mouse brains (red boxes). **B** Comparison of the respective CNS region sizes as percent of total brain volume. **C** Representative western blot for NfL expression in the respective CNS regions and spleen; β-Tubulin as control. **D** Quantification of the results shown in **C**. One-way ANOVA with Dunnett’s multiple hypothesis test. n ≥ 3. **E** Experimental workflow: mice were randomly assigned to an early (stable neuro-score 3 days after peak) and a late cohort (stable neuro-score 20 days after peak). **F** Graph depicting the mean and SEM EAE scores. n = 18. Specific time-points are indicated in red. **G** Linear regression and Pearson correlation of serum NfL (sNfL) and CSF NfL levels of early and late EAE combined. n = 18. **H** Pearson correlation of serum NfL (sNfL) and CSF NfL levels with the EAE score. n = 14. Images were created with BioRender and servier medical art. **I** Bar graph showing sNfL levels measured with SiMoA in pg/ml of early and late chronic EAE (n = 8 per condition, two-tailed Student’s t-test). *p < 0.05, **p < 0.01, ***p < 0.001

To determine the abundance of NfL in various murine brain regions in relation to their respective sizes, we first analyzed homogenates from forebrain, cerebellum, brainstem, optic nerve (ON) and SC for NfL protein expression (Fig. 1A). Immunoblotting revealed a high abundance of NfL in ON, SC and brain stem in comparison to the forebrain, despite their relatively small volumes (Fig. 1B–D). Thus, as expected, NfL expression is highest in brain structures with long, tightly packed axonal tracts, especially the SC. To investigate spatio-temporal NfL release in EAE, mice were randomly assigned to an early or late cohort (Fig. 1E, F). In pwMS, sNfL correlates well with NfL levels in the CSF (cNfL) [1]; sNfL levels are associated with disease progression (EDSS) and increase upon acute relapses and generally decline in remission [1]. Similarly, in our EAE model, sNfL levels showed a strong correlation with cNfL levels (Pearson’s *r* = 0.82, *p* < *0.001*), as well as EAE scores (*r* = 0.84, *p* < *0.001*) (Fig. 1G, H). Furthermore, in line with the reduction during remission in pwMS, sNfL levels were significantly higher in early than in late EAE (Fig. 1I). Taken together, these results demonstrate that MOG-EAE represents a suitable model to study NfL release from damaged axonal structures. Furthermore, NfL is highly abundant in the murine SC and sNfL levels behave similarly in EAE and pwMS.

### Spatio-temporal association of lesion localization and sNfL levels

**Fig. 2:**
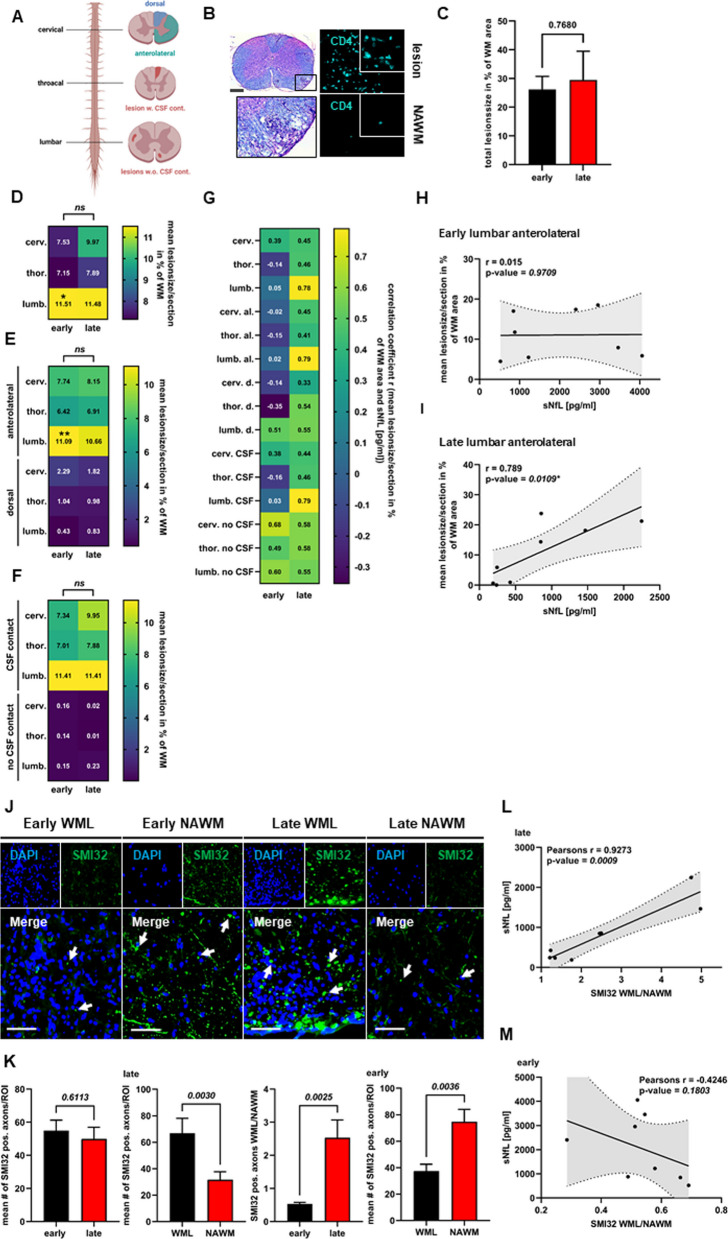
WML distribution in early and late MOG-EAE and differential axonal injury in early and late chronic MOG-EAE.** A** Cartoon depicting the analytical strategy of SC sections and anatomical classification of WML, with (w.) and without (w.o.) CSF contact (cont.). **B** Left: representative SC section stained with luxol fast blue (LFB) and H&E. Scale bar: 500 µm. Black box indicates an anterolateral WML with contact to CSF or pia. Right: CD4 staining in WML and NAWM. **C** Mean total WML sizes in percent of total white matter area per section from early and late chronic MOG-EAE (n = 8 per condition, two-tailed Student’s t-test). **D–F** Heat maps depicting the distribution of WML sizes in early and late MOG-EAE with respect to **D** the anatomical location (cervical, thoracic or lumbar); **E** the anatomical WML location in anterolateral or dorsal areas; and **F** the anatomical WML location and contact to the CSF or pia. One-way ANOVA with post hoc Dunnet ‘s test (n = 8 per condition). **G** Heat map depicting the association of sNfL levels with anatomical WML locations in early and late MOG-EAE (n = 8 per condition, Spearman’s rank correlation). **H–I** Correlation of sNfL levels with lumbar anterolateral WML locations in **H** early and **I** late chronic MOG-EAE (n = 8 per condition, Spearman’s rank correlation). *p < 0.05, **p < 0.01. **J** Representative images of SC WML and NAWM of MOG-EAE mice in early and late EAE. SMI32-positive axons (arrows) indicate damaged axons. DAPI staining indicates the inflammatory infiltrate. 40 × magnification. Scale bar: 50 µm. **K** Quantification of mean SMI32-positive axons and WML/NAWM per 200 × 200 µm region of interest (ROI) in early and late chronic EAE (n = 8 mice per condition). Two-tailed Student’s t-test and Mann–Whitney U. Mean and SEM. **L, M** Association of sNfL levels with axon WML/NAWM ratios in **F** early chronic and **G** late chronic MOG-EAE. Pearson’s rank correlation (n = 8 per group)

To determine the spatio-temporal origins of NfL, we first investigated the anatomical distribution of inflammatory white matter lesions (WML) in luxol- and H&E-stained SC sections from early and late MOG-EAE. WML were categorized according to their respective anatomical location (cervical, thoracic, lumbar, anterolateral or dorsal) and contact to the CSF (Fig. 2A, B). As expected, the size of WML did not differ between early and late EAE (Fig. 2C). In line with previous publications [6], WML showed a predominately lumbar distribution; however, this only reached statistical significance in the early EAE cohort (Fig. 2D). Furthermore, at both time-points, WML were mainly located in the lumbar, anterolateral SC and were in contact with the CSF or pia (Fig. 2D–F). In addition, there were few WML with no contact to the CSF or pia (Fig. 2F). Overall, early and late MOG-EAE are thus comparable with regard to the respective anatomical locations, sizes and overall number of WML.

Next, we sought to investigate the association of sNfL levels with size and number of WML in the respective anatomical locations at both time-points. Surprisingly, we did not find any significant correlation between WML size and location in early MOG-EAE (Fig. 2G, H). In contrast, in late MOG-EAE, sNfL levels were strongly associated with almost all WML locations (Fig. 2G, I). Best correlations were found for anterolateral lumbar lesions with contact to the CSF or pia (*r* = 0.789; *p* = *0.0109*) (Fig. 2G, I). Interestingly, there was no significant correlation between sNfL and WML with no contact to the CSF or pia (Fig. 2G). Consistent results were obtained for the number of WML (data not shown). Together, these results suggest that despite similar WML sizes, numbers and overall anatomical distribution, sNfL does not primarily originate from WML in early EAE and may arise from injured axons within the NAWM. In contrast, in late EAE, sNfL mainly arises from focal WML.

### Differential contribution of focal and diffuse axonal injury to NfL levels in early and late MOG-EAE

Next, we hypothesized that the observed spatio-temporal differences in the association of sNfL with WML may be caused by a differential pattern of neuroaxonal damage in early and late EAE. Therefore, we compared the number of injured (SMI32 +) axons in WML and NAWM at both time-points. In accordance with the observed lower sNfL levels in late EAE (Fig. 1H), the total number of SMI32 + axons per ROI in early and late EAE showed a non-significant trend towards the early time point (Fig. 2J, K; 54.9 vs. 49.9; *p* = *0.6113*). SMI32 stainings in early EAE revealed widespread axonal damage in NAWM, whereas there were significantly fewer damaged axons in WML (Fig. 2J, K; 70.7 vs. 37.4; *p* = *0.0036*). In contrast, in late EAE, axonal damage was mainly confined to WML, with significantly fewer injured axons in the NAWM (Fig. 2J, K; 66.8 vs. 31.7; *p* = *0.0152*). These spatio-temporal differences were particularly evident after comparing SMI32-lesion/NAWM ratios in early and late MOG-EAE (Fig. 2K; 0.54 vs. 2.53; *p* = *0.0025*). Moreover, in late EAE sNfL levels were strongly associated with SMI32-lesion/NAWM ratios (Fig. 2L, Pearson’s *r* = 0.9273; *p* = *0.0009*); in early EAE there was no significant association (Fig. 2M, r = − 0.4246; *p* = *0.1803*). Taken together, these results suggest that in EAE, neuroaxonal damage may evolve in a spatio-temporal direction from NAWM at early to WML in late disease stages.

### Spatio-temporal transcriptome analysis distinguishes focal and diffuse white matter damage in MOG-EAE

**Fig. 3:**
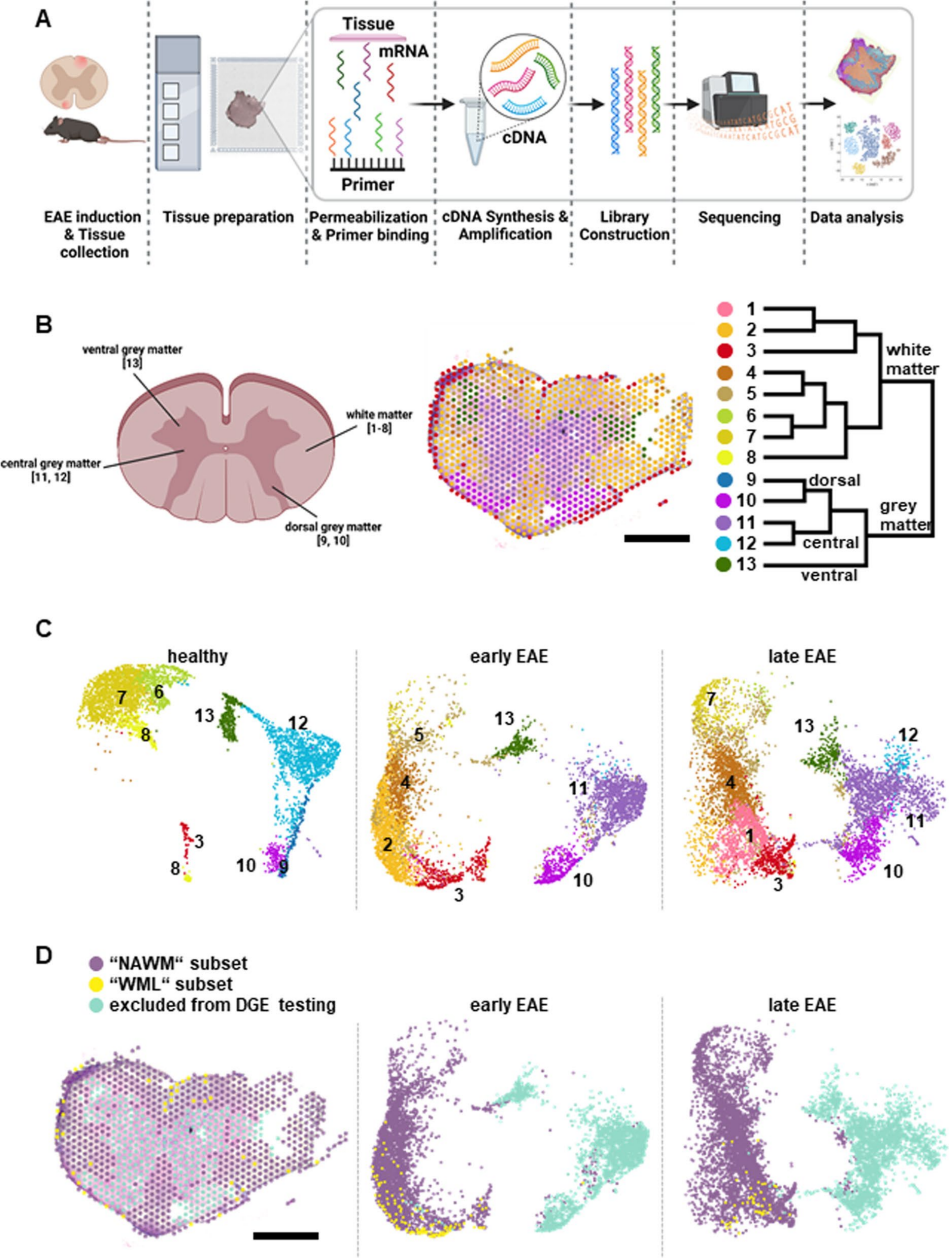
Spatial transcriptomics in MOG-EAE.** A** Experimental workflow of barcode-based spatial transcriptomics. Images were created with BioRender. **B** Cartoon depicting the anatomical location of barcoded spots in the clustering and in **C**. Clustering shows the spots color-coded according to the Seurat clustering (graph-based clustering and hierarchical cluster merging). Images were created with Loupe Browser. Scale bar: 1 mm. **C** UMAP visualization of all spots, split by condition: healthy (n = 2), early EAE and late EAE (n = 3, per condition). Seurat clusters are color-coded, labeled and annotated (in groups) by their anatomical location. Image created with Loupe Browser. **D** Subsets show the color-coding according to the two subsets for DGE-testing: “NAWM” (purple) and “WML” (yellow) and the excluded non-white-matter spots (teal). UMAP visualization of all spots split by condition: early EAE and late EAE (n = 3, per condition). Seurat clusters are color-coded: NAWM” (purple) and “WML” (yellow)

Next, we sought to gain insight into specific biological processes involved in the spatio-temporal differences of neuroaxonal damage and NfL release, by applying spatial RNA sequencing (spRNAseq). To this aim, we compared the spatial transcriptomes of NAWM and WML from early and late MOG-EAE (Fig. 3A). Overall, we identified 13 clusters in both early and late EAE (Fig. 3B, C); clusters 9–13 could be attributed to grey matter structures, whereas clusters 1–8 were associated with the white matter (Fig. 3B, D). Importantly, clusters 1–3 were associated with WML and were nearly exclusive for EAE (Fig. 3D). In total, we identified 134 differentially expressed genes (between early and late EAE) in WML and 1497 in NAWM (Additional file 1: Fig. A1A–C). Overall, 129 genes were significantly regulated in both subsets, 67 genes were regulated in both subsets, but only significant in NAWM.

**Fig. 4:**
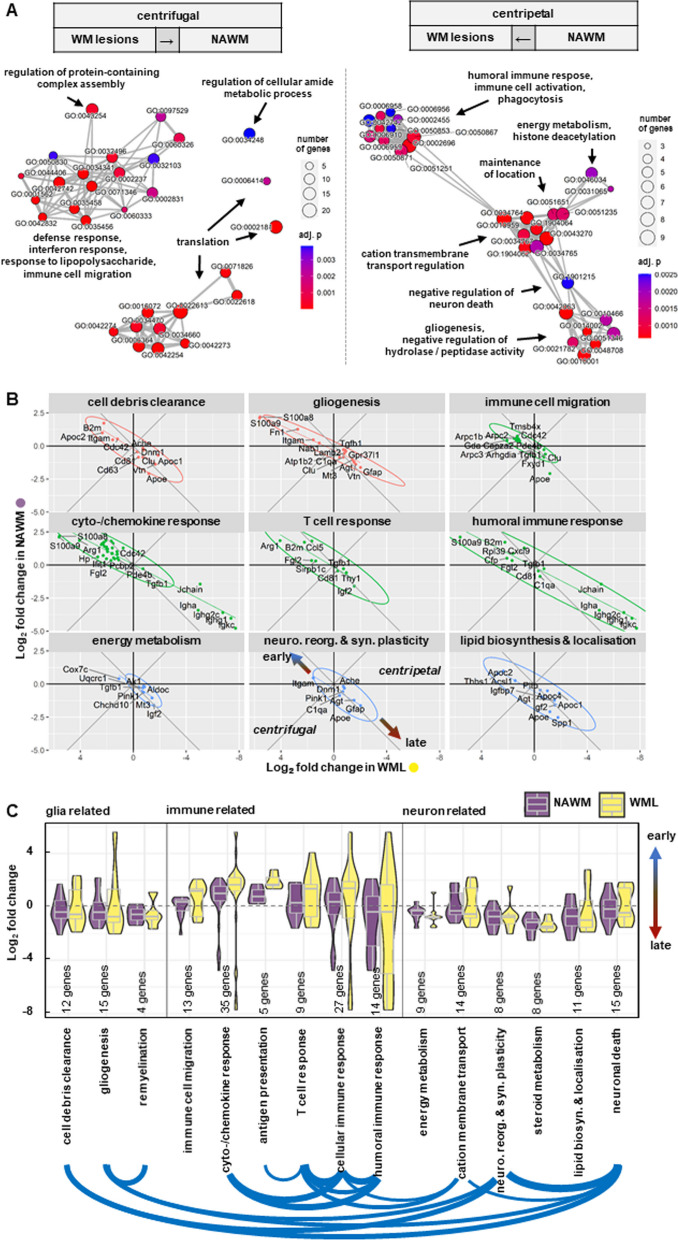
Comparison of temporal gene regulation of EAE processes between NAWM and WML using spatial transcriptomics.** A** GO term analysis results of DE genes that have a higher (left) or lower (right) fold change in “WML” than in “NAWM” and have *p* < 0.05 in both subsets and Bonferroni-corrected *p* < 0.05 in at least one. Networks of “biological processes” GO terms are plotted by gene set overlap (width of grey lines scaled by Jaccard index). Images were created with R package enrichplot. **B** Log_2_-fold changes from early-vs-late DGE-testing in “NAWM” (y axis) and “WML” (x axis) subset. Common DE genes between both subsets enriched in selected GO terms are plotted separately per functional modules with ellipse and regression line. A selection of the modules in **C**, ellipses are colored according to the 3 groups of modules. **C** Log_2_-fold changes early-vs-late in the subsets “NAWM” and “WML”. Common DE genes between both subsets enriched in selected GO terms are plotted per functional modules of “biological processes” GO terms as box-and-whiskers in violin-plots. Genes may appear in multiple modules. The modules were grouped into glia-related, immune-related, or neuron-related processes. Under the modules the pairwise overlap of their gene sets is drawn as blue arcs (line width scaled by Jaccard index, minimum 0.15)

A GO term network analysis including all genes that were regulated in both subsets revealed a centrifugal pattern (from WML towards NAWM) for GO terms of cytokine/interferon response, immune cell migration, defense- and lipopolysaccharide response (Fig. 4A) and a centripetal pattern (from NAWM towards WML) for GO terms of gliogenesis, regulation of cation transmembrane transport and humoral adaptive immune response/phagocytosis (Fig. 4A). Next, in order to identify specific spatio-temporal differences of gene expression between early and late EAE, we used GO term enrichment analysis on significantly regulated genes. We summarized network clusters from enrichment analysis GO terms as 15 modules of immune-, glia- and neuron-related processes and used them to annotate genes that were regulated in both white matter subsets (Fig. 4B, C; Additional file 1: Fig. A2). Overall, genes annotated for glia cell-related processes, such as cell debris clearance (*Apoe, Cd63, Cd81, Clu, Vtn, Apoc1, Apoc2, Dnm1, Cdc42, Itgam*), gliogenesis (*Gfap, Agt, Clu, C1qa, Atp1b2, Mt3, Vtn, Tgfb1, Lamb2, and Gpr37l1*) and (re-) myelination (*Clu, Vtn and Tgfb1*), as well as neuron-related processes like neuronal reorganization and plasticity (*Apoe, Gfap, Agt, C1qa, Pink1, Dnm1 and Ache*) were upregulated in late EAE (Fig. 4B; Additional file 1: Fig. A1). Humoral immune response- (*Igkc, B2m, Ighg1, Igha, Cxcl9, Ighg2c, Jchain, S100a9, Cd81, Tgfb1, Rpl39, Cfp and Fgl2*) and neuronal death-associated modules (*Apoe, Ccl5, Lcn2, Agt, Clu, Mt3, C1qa, Pink1, Npm1, Ccr5, Hsp90ab1, Cdc42, Ache, Serpinf1 and Itgam*) showed comparable levels in both early and late EAE, whereas genes associated with T cell response (*B2m, Ccl5, Arg1, Sirpb1c and Fgl2*), cyto-/chemokine response (*Gbp2, B2m, Irgm1, Igtp, Iigp1, Ccl5, Gbp6, Stat1, Ifi47, Lcn2, Ifi27l2a, Gbp7, Cxcl9, H2-K1, Irgm2 and Arg1*) and antigen presentation (*B2m, H2-K1, Thbs1, Rab32 and Fgl2*) were upregulated in early EAE (Additional file 1: Fig. A2). Especially genes linked to interferon response/signaling (*Gbp6, Gbp7, Ifi27l2a, Irgm1, Igtp, Ifi47, and Stat1*) were upregulated in early WML (Additional file 1: Fig. A1B). Interestingly, the only early regulated genes, stronger regulated in NAWM compared to WML were genes predominately produced by myeloid cells upon stimulation with IFNγ (*Cxcl9, Ccl5 and Lcn2*) (Fig. 4B) [23]. Together, these results suggest that the early phase of EAE is largely driven by cyto-/chemokine secretion, antigen presentation and infiltration of immune cells, whereas late EAE is characterized by an accumulation of glial cells within WML. Therefore, the distribution of especially glial cell processes coincides with our identified patterns of neuroaxonal damage in late EAE.

### Association between myeloid cell infiltration and neuroaxonal damage

**Fig. 5:**
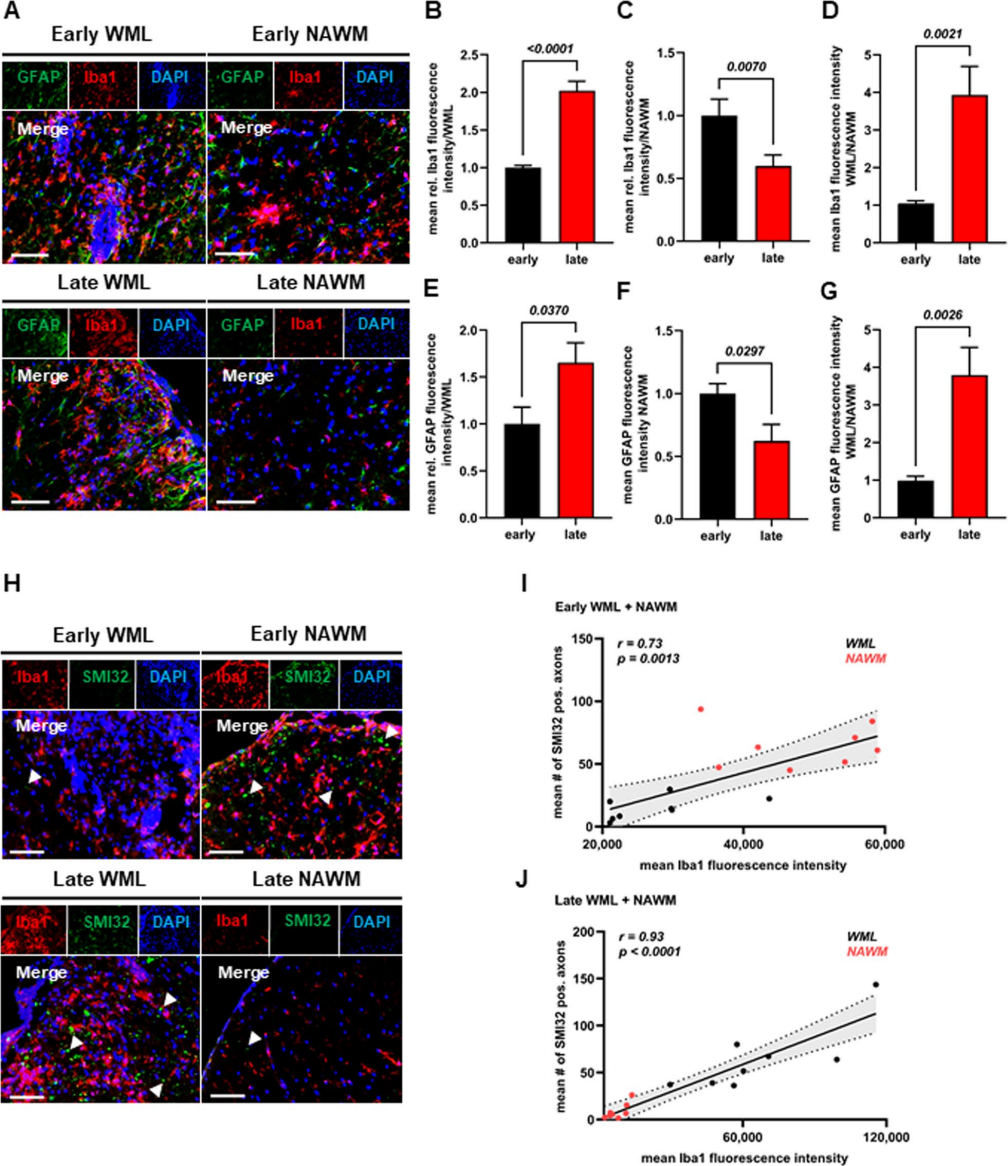
Association between glial cell accumulation and neuroaxonal damage. **A** Representative images of SC WML and NAWM of MOG-EAE mice in early and late EAE. Iba1 and GFAP staining. DAPI staining indicates the inflammatory infiltrate. 40 × magnification. Scale bar: 50 µm. **B–D** Quantification of mean iba1 fluorescence intensity in **B** WML and **C** NAWM as well as **D** mean iba1 fluorescence intensity ratios of WML/NAWM in early and late EAE (n = 8 mice per condition). 2-tailed Student’s t-test or Mann–Whitney U. Mean and SEM. **E–G** Quantification of mean GFAP fluorescence intensity in **E** WML and **F** NAWM as well as **G** mean GFAP fluorescence intensity ratios of WML/NAWM in early and late EAE (n = 8 mice per condition). 2-tailed Student’s t-test. Mean and SEM. **H** Representative images of SC lesions and NAWM of MOG-EAE mice in early and late EAE. Iba1 and SMI32 staining. DAPI staining indicates the inflammatory infiltrate. 40 × magnification. Scale bar: 50 µm. Arrowheads indicate microglial cells in close contact with axons. **I, J** Association of iba1 fluorescence intensity and mean numbers of SMI32-positive axons in **I** early and **J** late EAE (n = 8 per condition, Spearman or Pearson correlation)

Based on the spRNAseq results, we hypothesized that the observed spatio-temporal patterns of axonal damage may result from a differential glial cell infiltration. Thus, we performed immunostainings of CMC (Iba1 + cells) and astrocytes (GFAP + cells) in lesions and NAWM. Following the spatial pattern of axonal damage at both time-points (Fig. 2), Iba1/GFAP stainings revealed a twofold increase in CMC (Fig. 5A, B; *p* < *0.0001*) and a 1.7-fold increase in astrocytes in late WML (Fig. 5A, E; *p* = *0.0370*). In contrast, in NAWM, we observed a 0.4-fold decrease in both CMC (Fig. 5A, C; *p* = *0.0230*) and astrocytes in late- as compared to early EAE (Fig. 5A, F; *p* = *0.0297*). Again, these spatio-temporal differences were particularly evident by comparing Iba1- (Fig. 5D; fourfold increase in late EAE; *p* = *0.0021*) and GFAP-lesion/NAWM ratios (Fig. 5G; fourfold increase in late EAE; *p* = *0.0026*). Hence, CMC and astrocyte infiltrates follow the respective spatio-temporal pattern of neuroaxonal damage in MOG-EAE. Especially CMC have been implicated in promoting neuroaxonal damage in pwMS [7, 24]; therefore, we next asked whether neuroaxonal damage (SMI32 + axons) is associated with the abundance of CMC (Iba1 + cells) in both early and late EAE. Indeed, we observed a strong correlation of injured axons (SMI32 +) and CMC in both early (Fig. 5H, I; Spearman’s *r* = 0.77; *p* = *0.0008*) and late EAE (Fig. 5H, J *r* = 0.93; *p* < *0.0001*). Taken together, these results suggest that neuroaxonal damage in MOG-EAE can be mediated by CMC and both processes may progress in a disease stage-dependent centripetal manner from NAWM to WML.

### Microglia activation mediates NfL release from damaged axons

**Fig. 6:**
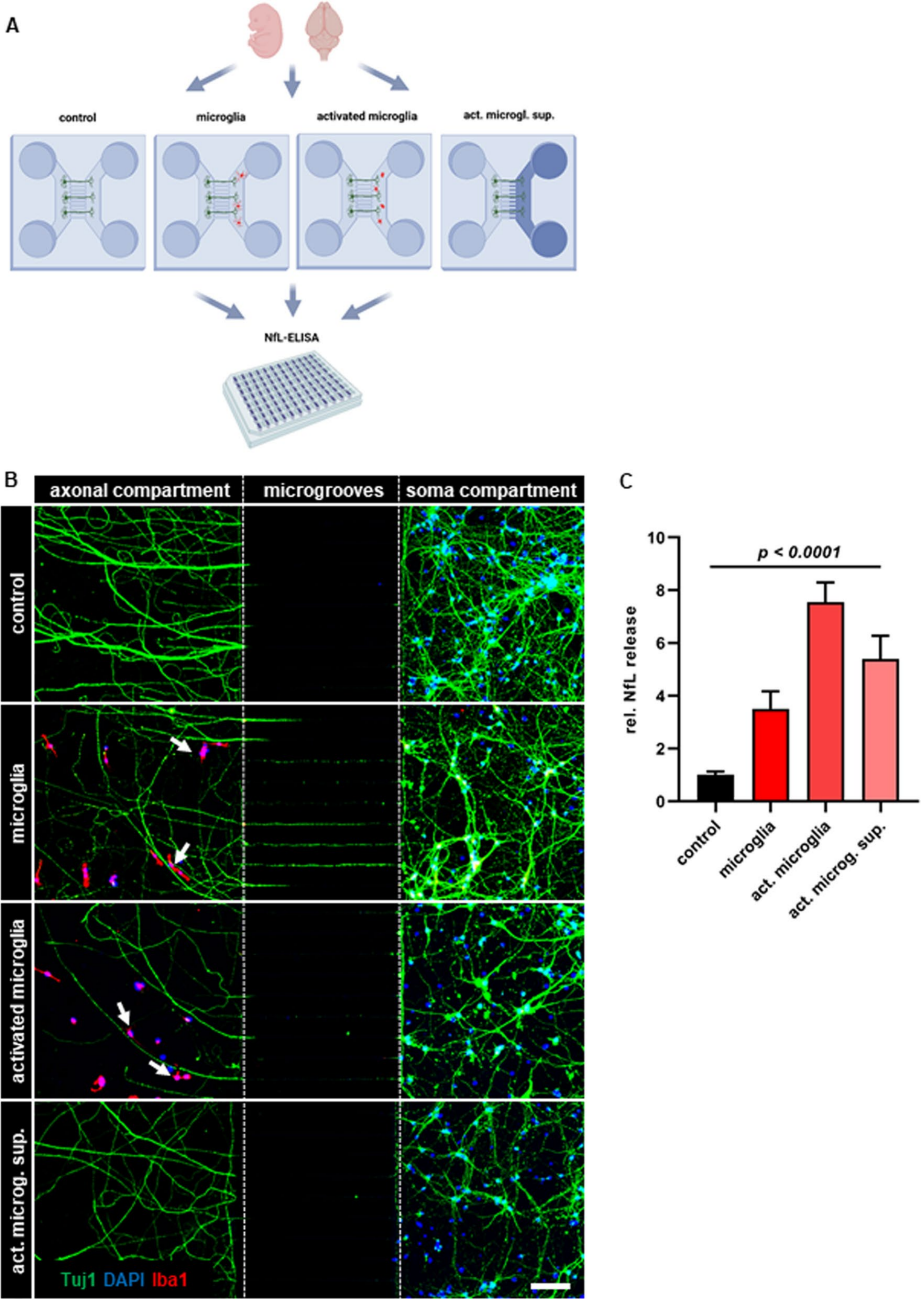
Co-cultures of neuroaxons and CNS-myeloid cells.** A** Cartoon depicting the experimental workflow of neuroaxonal-CMC co-cultures. Act. microgl. sup.: activated microglia supernatant. Image was created with BioRender. **B** Representative images of neuroaxonal-CMC co-cultures. Neurons were stained with Tuj1 (green), CMCs were stained with Iba1; nuclear counter-stain with DAPI. Scale bar: 100 µm. Arrows indicate microglia in close contact with axons. **C** Quantification of NfL release in neuroaxonal-CMC co-culture supernatants, relative to control (neuron culture medium) (n = 6–10, per condition). One-way ANOVA with post hoc Dunnet’s test. Mean and SEM

In accordance with our data, CMCs, such as microglia, have been associated with neuroaxonal damage in many primary and secondary neurodegenerative disorders, including MS [7]; however, it is unclear whether they can directly prompt soluble NfL release from axonal structures. Especially in WML, microglia get in close proximity with axonal tracts and not the respective neuronal cell bodies. Thus, in order to investigate microglia-mediated NfL release, we established a microglia–axon co-culture system in which axons from dissociated primary neurons were separated from the soma compartment and brought into contact with naïve, LPS-activated or conditioned media from LPS-activated primary microglia (Fig. 6A, B). “Naïve” microglia increased NfL release about 3.5-fold (Fig. 6C; one-way ANOVA; *p* < 0.0001), whereas LPS-activated microglia increased NfL secretion about 7.5-fold (Fig. 6C; *p* < 0.0001). Conditioned medium from LPS-activated microglia alone increased NfL release by about sixfold (Fig. 6C; *p* < 0.0001). In line with elevated NfL levels, we observed a concomitant degradation of the neuronal network, both in the axonal and soma compartments (Fig. 6B). Thus, microglia are not only associated with neuroaxonal damage, but can directly cause NfL release from damaged axons through both cell–cell contact and the release of soluble factors.

## Discussion

Implementation of NfL into clinical decision-making requires a deeper understanding of underlying pathophysiological pathways of NfL release. Importantly, factors such as changes over specific disease stages or contribution of anatomical locations can best be addressed by experimental approaches. Here, we present the first systematic animal study demonstrating the spatio-temporal and pathophysiological origins of sNfL in neuroinflammation, applying SiMoA, immunohistochemistry and spatial transcriptomics. While sNfL showed no correlation with WML in early EAE, therefore indicating little neuronal damage, highest sNfL release and correlations were identified for anterolateral lumbar lesions with contact to the CSF or pia in late EAE. It remains unclear why lesions with contact to the CSF or pia correlate better with sNfL levels than those without contact. However, we identified very few lesions without contact to the CSF or pia overall, and it is possible that in lesions with contact to the CSF or pia, NfL may have quicker access to the subarachnoid space and potential CSF-draining routes, such as meningeal lymphatics [25, 26].

NfL is a major structural component of the axonal cytoskeleton, with larger myelinated axons containing more NfL than smaller axons [3, 4]. The anterolateral murine SC contains several tracts with large caliber axons, such as the spinothalamic and -cerebellar, as well as parts of the corticospinal tract [27]. Therefore, our results are in line with the high abundance of NfL in such large axonal tracts and may explain why patients with SC lesions show higher sNfL levels [3–5]. Moreover, despite lower abundance, dorsal WML also displayed good correlations with sNfL levels, as the dorsal column also contains large caliber axons running in the corticospinal tract, as well as the gracile and cuneate fascicle [27]. Therefore, our study indicates that the magnitude of NfL release is indeed dependent on the respective anatomical location of inflammatory CNS lesions in late EAE. Thus, high intra-individual variances in sNfL levels in pwMS may partly result from the specific lesion locations (damage to long tracts of large caliber axons), but also from differences in the involvement of the NAWM. In accordance with post-mortem studies [28, 29], our results demonstrate that there is a substantial amount of neuroaxonal damage within the NAWM. The concept of NAWM is primarily based on magnetic resonance imaging (MRI) as the principle diagnostic tool in MS. However, together with recent findings [28, 30], our results highlight the need for additional biomarkers, such as sNfL or sGFAP, which can aid MRI in identifying pathological processes in areas where MRI is functionally “blind”. Thus, future diagnostic work-ups may need to include combinations of multimodal biomarkers (such as MRI, optical coherence tomography and blood) thereby enabling more precise diagnosis, prognostication and therapeutic decision-making [13].

In fact, our spatial transcriptomic data outline a pathophysiological cascade of T cell infiltration and local antigen presentation in both WML and NAWM during early EAE [31, 32]. Widespread cyto-/chemokine secretion by invading T cells may not only attract further leukocytes, but also activate bystander CMC and astrocytes, causing neuroaxonal damage and therefore extensive NfL release, especially within the NAWM. Recent findings have highlighted alterations of the axon–myelin unit accompanied by an increased presence of activated and phagocytic CMC and T cells in MS NAWM [28]. Indeed, we identified pronounced neuroaxonal damage and accumulation of CMC in NAWM of early EAE. Furthermore, the main differentially regulated genes in early NAWM were Cxcl9 and Ccl5, both chemokines predominately produced by myeloid cells upon stimulation with interferon gamma (IFNγ) [23]. Interestingly, especially IFNγ signaling was upregulated in our transcriptomic data set in early EAE. IFNγ has not only been shown to damage oligodendrocytes [33], but may also attract CMC to sites of accumulating cell debris via the CXCR3 signaling pathway [34]. Therefore, IFNγ secretion in early EAE may drive the subsequent CMC accumulation in late WML. Furthermore, we identified clusters of lipid biosynthesis and steroid/cholesterol metabolism in late WML, which may reflect the need of CMC to phagocytose and process lipid-rich myelin debris, and to produce precursors for secondary messengers [35].

Together with CMCs, we identified a concomitant accumulation of astrocytes (GFAP + cells) in late WML. Thus, as recent examples demonstrate, it is likely that together with CMC, astrocytes form a proinflammatory neurodegenerative niche in which their chronic activation, e.g., by humoral factors, creates an environment of smoldering neuroaxonal degeneration [8]. Strikingly, our transcriptomic data revealed a humoral immune response cluster in late WML, in which especially immunoglobins were the strongest differentially regulated genes (Fig. 5B). IgG and activated complement have been found in MS lesions and IgG immune complexes strongly potentiate the activation of CMC [8, 36]. In accordance with the increased neuroaxonal damage in late WML, spatial transcriptomics identified gene clusters indicative of a high-energy demand and mitochondrial redistribution in degenerating axonal structures [28].

One could argue that other cell types, such as encephalitogenic T cells, may have also driven neuroaxonal damage and concomitant NfL release in our study and CMC may merely be bystanders of a T cell-driven neurotoxicity. In fact, encephalitogenic T cells have been shown to exert noxious effects through cell–cell interactions with neurons and oligodendrocytes, as well as the release of neurotoxic humoral factors [37–39]. However, T cells also interact with or act through CNS-resident cells and there is a large body of evidence supporting a neurodegenerative role of CMC in MS and EAE. First, our axon–microglia co-cultures clearly demonstrate that CMC (especially activated CMC) can directly damage neuroaxons and induce NfL release. Activated CMC have been shown to promote neuronal damage by secretion of proinflammatory cytokines (tumor necrosis factor alpha [TNFα], interleukin-1 beta [IL1β]), biolipids, reactive oxygen and nitrogen species, as well as release of proteases and even glutamate [24, 40]. Furthermore, there is some recent evidence from human post-mortem studies demonstrating a close association of CMC activation and neurodegeneration [7, 8, 28, 29]. Moreover, in relapsing–remitting MS patients, NfL and YKL-40 (a marker of microglial activation) correlate well with each other [41, 42].

Taking these findings into account, inhibition of CMC may be a viable strategy to mitigate neuroaxonal damage already at early stages of MS. However, it is important to note that CMC are also capable of defending the CNS from autoreactive lymphocytes, e.g., by capturing and engulfing invading proinflammatory T cells during neuroinflammation or interacting with regulatory T cell populations [10, 43]. Furthermore, regenerative CMC subtypes can also promote remyelination of denuded axons by clearing accumulated myelin debris [44]. In accordance with this, we identified genes associated with clearance of cell debris, remyelination and lipoprotein complex remodeling, particularly in late EAE. Therefore, a delicate balance between CMC inhibition and necessary activation may be required in order to facilitate optimal therapeutic outcomes.

BTK inhibitors can reduce the activation and proliferation, as well as the production of proinflammatory cytokines by CMC and have shown first promising results in two recent phase II clinical trials [45, 46]. However, taking the different roles of CMCs in MS into account, further research is needed to fully understand the effect of BTK inhibitors on CMC and other immune cells within the CNS.

The main limitation of our study is the lack of patient data. However, our results integrate well with recent post-mortem studies and we have shown that sNfL behaves in a comparable way in EAE as it does in pwMS. Therefore, EAE can serve as a valid model to study inflammatory neurodegeneration and specifically NfL biology.

Taken together, our data demonstrate that neuroaxonal damage and sNfL release in MOG-EAE can be driven by CMC and may further progress in a disease stage-dependent centripetal pattern from NAWM to WML.

### Supplementary Information

**Additional file 1: Figure A1. **Differential gene expression between early and late EAE in NAWM and WML. **Figure A2**. Comparison of temporal gene regulation of EAE processes between NAWM and WML using spatial transcriptomics.

## Data Availability

The data supporting this study are available upon reasonable request. Raw data from RNA-sequencing have been deposited in NCBI’s Gene Expression Omnibus under the following accession code: GSE226591. A password to access the data is available upon reasonable request.

## Abbreviations

CMC: CNS-myeloid cells
CSF: Cerebrospinal fluid
EAE: Experimental autoimmune encephalomyelitis
EDSS: Expanded disability status scale
MOG: Myelin oligodendrocyte glycoprotein
NAWM: Normal-appearing white matter
SiMoA: Single molecule array
SC: Spinal cord
sNfL: Serum neurofilament light chain
WML: White matter lesions
