# Supplementary material for: Spatial transcriptomics and neurofilament light chain reveal changes in lesion patterns in murine autoimmune neuroinflammation
Source: J Neuroinflammation. 2023 Nov 13;20:262. doi: 10.1186/s12974-023-02947-y (PMC10644497; doi:10.1186/s12974-023-02947-y)
Supplement: Supplementary file 1 — Additional file 1: Figure A1. Differential gene expression between early and late EAE in NAWM and WML. Figure A2. Comparison of temporal gene regulation of EAE processes between NAWM and WML using spatial transcriptomics. [file 12974_2023_2947_MOESM1_ESM.pdf]

Additional file 1

Spatial transcriptomics and neurofilament light chain reveal changes in lesion patterns in murine autoimmune neuroinflammation

Tobias Brummer[1] MD, Miriam Schillner[1] PhD, Falk Steffen[1] MD, Flores Kneilmann[1] MSc, Beatrice Wasser PhD[1], Timo Uphaus[1] MD, Frauke Zipp[1] MD, Stefan Bittner[1]* MD

[1] Department of Neurology, Focus Program Translational Neuroscience (FTN) and Immunotherapy (FZI), Rhine Main Neuroscience Network (rmn[2]), University Medical Center of the Johannes Gutenberg University Mainz, Mainz, Germany

***Correspondence**
Stefan Bittner
Department of Neurology, Focus Program Translational Neuroscience (FTN) and Immunotherapy (FZI), Rhine Main Neuroscience Network (rmn[2]), University Medical Center of the Johannes Gutenberg University Mainz, Langenbeckstr. 1, 55131 Mainz, Germany
Tel.: +49-(0)6131-17-7156
E-mail address: bittner@uni-mainz.de

Additional Figures

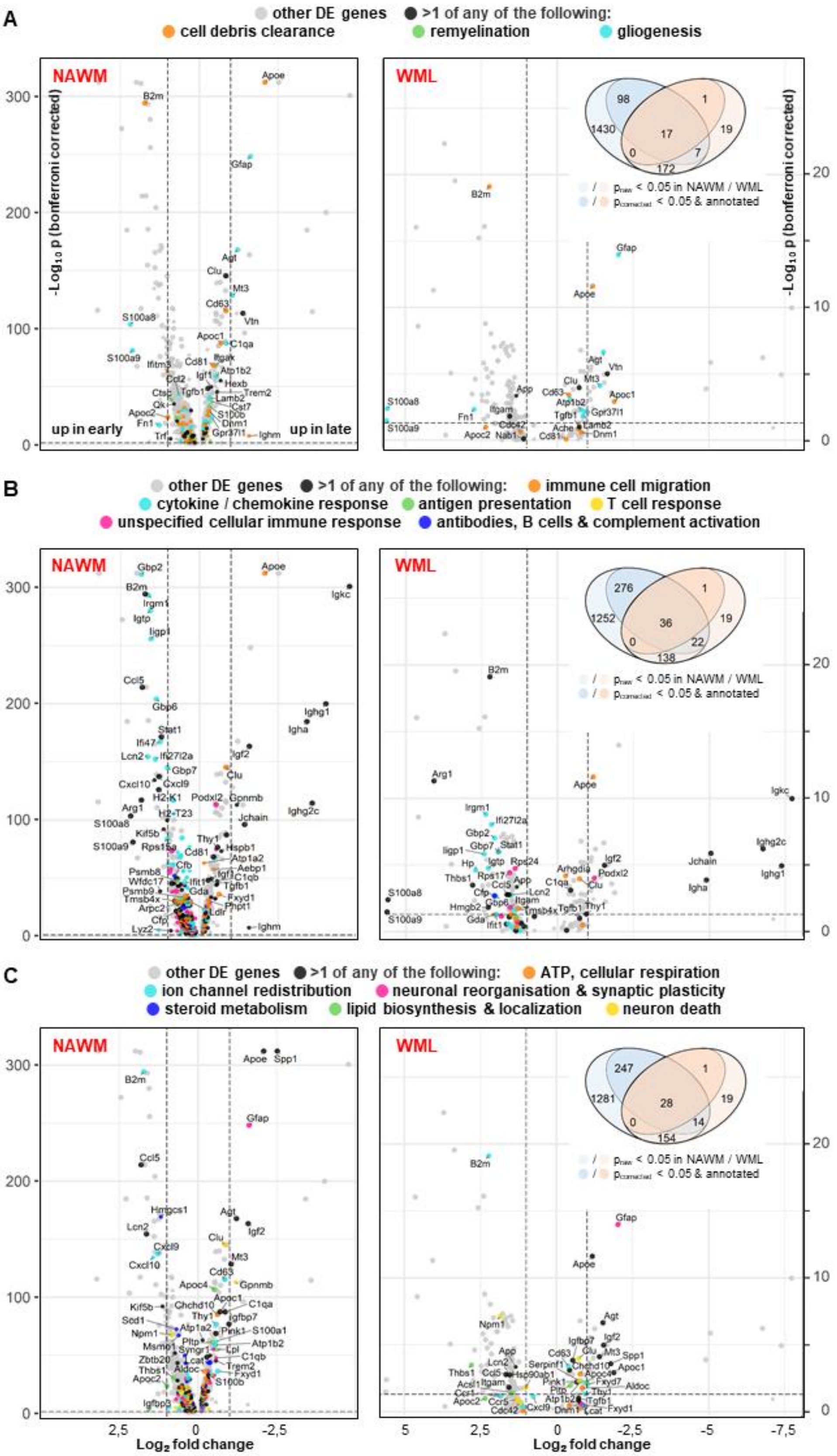

Figure A1. Differential gene expression between early and late EAE in NAWM and WML. Volcano plots of the results of early-vs-late DGE-testing of spatial transcriptomic Seq-data with MAST in the subsets “NAWM” and “WML”. DE genes from both

subsets enriched in selected GO-terms are color-coded per functional modules of "biological processes" GO-terms. Modules are grouped (A, B, C), in the same way as in Figure 6 (glia-related, immune-related, neuron-related, respectively), and volcano are plots shown separately for each labeling group. Log_2 fold change 1 and -1 and Bonferroni-corrected p of 0.05 are indicated by dashed lines. Genes that appear in both subset‘s DGE and have Bonferroni-corrected $p < 0.05$ in at least one of them are plotted with larger point size. Images were created with R package EnhancedVolcano. Venn diagrams indicate identified common genes in both subsets.

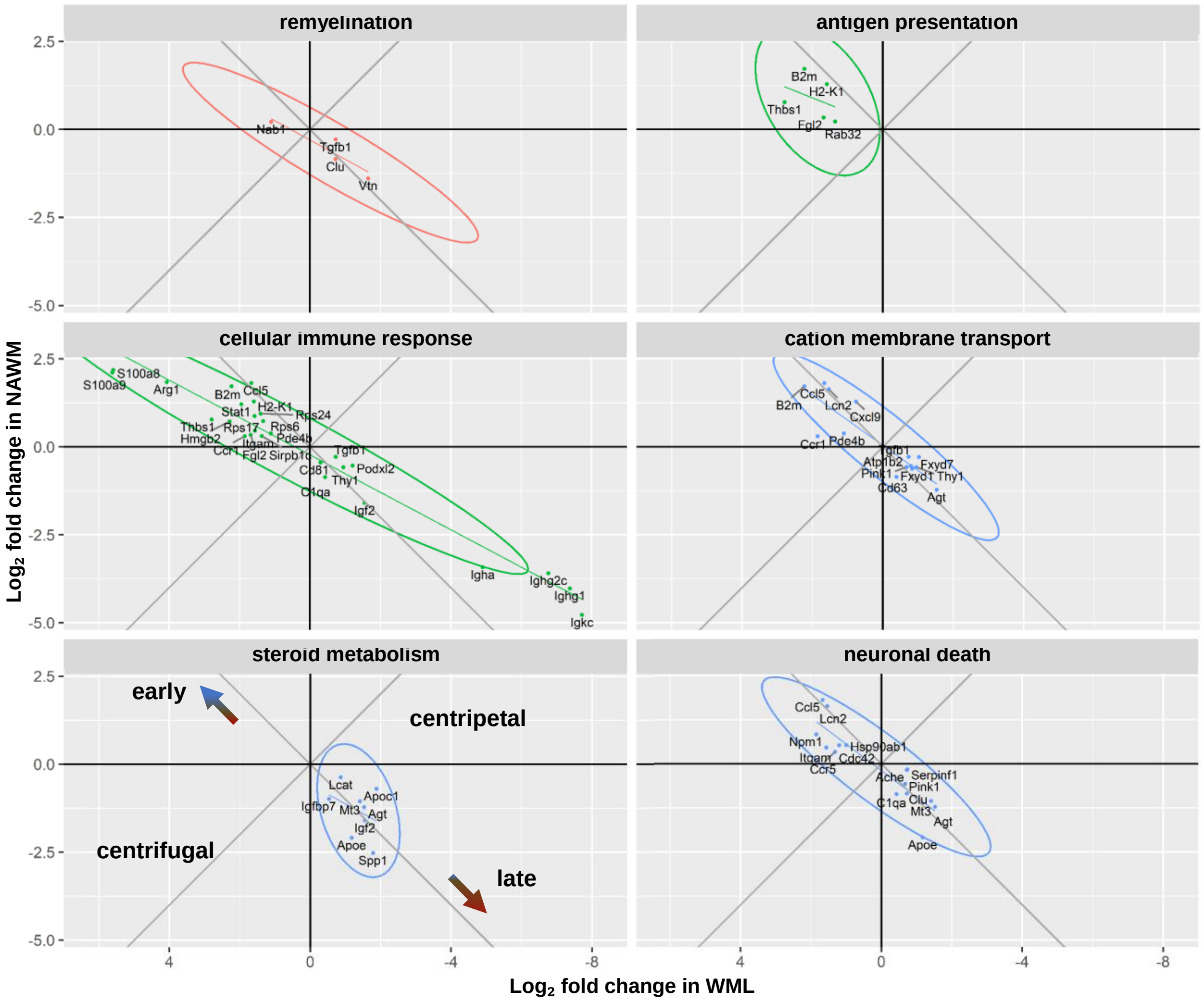

Figure A2. Comparison of temporal gene regulation of EAE processes between NAWM and WML using spatial transcriptomics Log_2 fold changes from early-vs-late DGE-testing in "NAWM" (y axis) and "WML" (x axis) subset. Common DE genes between both subsets enriched in selected GO terms are plotted separately per functional modules with ellipse and regression line.
